# Quantitative shear wave ultrasound elastography: initial experience in solid breast masses

**DOI:** 10.1186/bcr2787

**Published:** 2010-12-01

**Authors:** Andrew Evans, Patsy Whelehan, Kim Thomson, Denis McLean, Katrin Brauer, Colin Purdie, Lee Jordan, Lee Baker, Alastair Thompson

**Affiliations:** 1 Centre for Oncology and Molecular Medicine, University of Dundee, Ninewells Hospital & Medical School, Dundee DD1 9SY, UK; 2 Breast Imaging Department, Ninewells Hospital, Dundee DD1 9SY, UK; 3 Pathology Department, Ninewells Hospital, Dundee DD1 9SY, UK

## Abstract

**Introduction:**

Shear wave elastography is a new method of obtaining quantitative tissue elasticity data during breast ultrasound examinations. The aims of this study were (1) to determine the reproducibility of shear wave elastography (2) to correlate the elasticity values of a series of solid breast masses with histological findings and (3) to compare shear wave elastography with greyscale ultrasound for benign/malignant classification.

**Methods:**

Using the Aixplorer^® ^ultrasound system (SuperSonic Imagine, Aix en Provence, France), 53 solid breast lesions were identified in 52 consecutive patients. Two orthogonal elastography images were obtained of each lesion. Observers noted the mean elasticity values in regions of interest (ROI) placed over the stiffest areas on the two elastography images and a mean value was calculated for each lesion. A sub-set of 15 patients had two elastography images obtained by an additional operator. Reproducibility of observations was assessed between (1) two observers analysing the same pair of images and (2) findings from two pairs of images of the same lesion taken by two different operators. All lesions were subjected to percutaneous biopsy. Elastography measurements were correlated with histology results. After preliminary experience with 10 patients a mean elasticity cut off value of 50 kilopascals (kPa) was selected for benign/malignant differentiation. Greyscale images were classified according to the American College of Radiology (ACR) Breast Imaging Reporting and Data System (BI-RADS). BI-RADS categories 1-3 were taken as benign while BI-RADS categories 4 and 5 were classified as malignant.

**Results:**

Twenty-three benign lesions and 30 cancers were diagnosed on histology. Measurement of mean elasticity yielded an intraclass correlation coefficient of 0.99 for two observers assessing the same pairs of elastography images. Analysis of images taken by two independent operators gave an intraclass correlation coefficient of 0.80. Shear wave elastography versus greyscale BI-RADS performance figures were sensitivity: 97% vs 87%, specificity: 83% vs 78%, positive predictive value (PPV): 88% vs 84%, negative predictive value (NPV): 95% vs 82% and accuracy: 91% vs 83% respectively. These differences were not statistically significant.

**Conclusions:**

Shear wave elastography gives quantitative and reproducible information on solid breast lesions with diagnostic accuracy at least as good as greyscale ultrasound with BI-RADS classification.

## Introduction

Greyscale ultrasound has a long-established role in the assessment of symptomatic breast masses, screen-detected abnormalities and the local staging of breast cancer [1]. Ultrasound is highly accurate in the benign/malignant differentiation of breast masses [2,3] and is useful in predicting the invasive extent of breast cancers in many cases [4]. Most solid breast masses still undergo percutaneous breast biopsy, however, usually under ultrasound guidance [5].

Assessment of anatomical structures by palpation in medical practice relies partly on perceived differences in tissue firmness. This property can be described by Young's Modulus, which is defined as:

E=σ/ε

where σ is the applied stress and ε is the resultant deformation of the tissue. This can also be termed stiffness or elasticity. Benign lesions tend to be soft, while malignant lesions tend to be firmer (that is, relatively stiff). Exceptions do occur - for example, mucinous cancers can be soft while postoperative scarring can be stiff. The stiffness of malignant lesions may be related to the desmoplastic reaction seen within and around many cancers.

Ultrasound static elastography provides a colour map of tissue elasticity that is superimposed on the real-time greyscale ultrasound image. Invasive breast cancers have been shown to be stiffer than benign or normal tissues [6]. Consequently, a number of scoring systems comparing the presence, size and distribution of areas of elasticity within the greyscale ultrasound abnormality have been devised [6,7]. Invasive cancers often produce areas of elasticity congruent with or appreciably larger than the greyscale abnormality [7,8]. A static elastography abnormality larger than the greyscale abnormality is highly suggestive of invasive malignancy [7]. Areas of ductal carcinoma *in situ *(DCIS) have static elastography values that are intermediate between those seen in invasive cancer and in fibroadenomas [7]. Static elastography has similar diagnostic performance to conventional greyscale ultrasound imaging, but breast static elastography has been hampered by significant interobserver variability [9,10]. Statistically significant differences are seen when comparing the area under receiver-operating characteristic (ROC) curve values from different observers, and computer-assisted quantification has been suggested as a means of overcoming such variability [11].

Shear wave elastography is a new method of obtaining elastography images based on the combination of a radiation force induced in a tissue by an ultrasonic beam and an ultrafast imaging sequence capable of catching in real time the propagation of the resulting shear waves [12,13]. The local shear wave velocity is recovered, enabling the production of a two-dimensional map of shear elasticity. The technique is performed using a conventional linear array probe and so can be incorporated into standard diagnostic ultrasound examinations [14].

The production of the radiation force by the probe rather than the operator (as applied in conventional ultrasound elastography) means shear wave elastography is more reproducible than conventional elastography. Within a given region of interest (ROI), defined by an electronic cursor, values for the maximum stiffness, mean stiffness and standard deviation (SD) are produced. Areas of stiffness can thus be clearly mapped. This reproducible, quantitative information is not available with standard elastography.

Until recently the published literature on shear wave elastography of the breast was limited to one small study consisting of 15 patients. This study demonstrated good separation of mean elasticity, measured in kilopascals, between fatty tissue (3 kPa), dense parenchyma (45 kPa), benign lesions (<80 kPa) and malignant lesions (>100 kPa) [14]. In July 2010 a paper was published indicating that shear wave elastography elasticity values were helpful in differentiating benign from malignant breast masses [15]. In this study, shear wave elastography had superior performance to Breast Imaging Reporting and Data System (BI-RADS) [16] classification as estimated from areas under ROC curves. It is possible that the addition of shear wave elastography may increase the ability of breast ultrasound to differentiate between benign and malignant masses. This may in turn allow a larger proportion of women with benign masses to be reassured than is currently possible following ultrasound examination, without the need for biopsy or short-term follow-up.

Our study aimed to determine the reproducibility of shear wave elastography findings as this issue was not addressed in previous studies. The present study also aimed to correlate a number of elasticity values (mean stiffness, maximum stiffness and SD) of a consecutive series of solid breast masses with percutaneous and surgical histology findings, and to investigate the accuracy of shear wave elastography compared with greyscale ultrasound with BI-RADS classification [16] in distinguishing benign from malignant breast lesions.

Previous studies have only addressed the significance of mean elasticity values.

## Materials and methods

The study group consisted of consecutive patients with solid lesions identified during routine breast scans using the Aixplorer^® ^ultrasound system (SuperSonic Imagine, Aix en Provence, France), which was installed in one of three ultrasound rooms within our breast imaging department. The probe used for the greyscale and shear wave elastography had a frequency range of 7.5 to 15 MHz, which at -6 dB gives axial resolution of 0.3 to 0.5 mm and lateral resolution of 0.3 to 0.6 mm.

Patients included women with symptoms and women with screen-detected abnormalities who were scanned by one of two breast radiologists or an advanced radiography practitioner trained to perform breast ultrasonography. These practitioners had between 5 and 20 years of breast ultrasound experience and work purely in breast imaging. The SuperSonic Imagine applications specialist was present for a week at the beginning of the study for practitioner training. Only women with lesions subjected to percutaneous biopsy were included in the study (women younger than 25 years old with clinically and sonographically benign legions do not undergo biopsy at our institution). In accordance with the applicable National Research Ethics Service guidance, ethical approval for the study was not required [17]. Written informed consent to use images was obtained, however, according to routine practice in our institution. Greyscale and elastography images were obtained within the standard ultrasound appointment. The combined echographic and elastographic examination time was between 10 and 20 minutes. Acquisition of the elastographic images added 3 to 5 minutes to the appointment time. The elasticity measurements were obtained by reviewing the images at a later time to ensure that the elastography examination did not interfere with clinic work flows.

At least two orthogonal greyscale images were obtained of each solid lesion using the same equipment used to perform the shear wave elastography. These images underwent BI-RADS [16] classification by a breast radiologist who was blinded to the elastography findings. BI-RADS categories 1 to 3 were taken as benign, since the American College of Radiology guidelines state that such lesions can be managed without immediate biopsy. BI-RADS scores of 4 or 5 were taken as malignant.

Two orthogonal elastography images were obtained for each of the lesions. Although obtaining elastography images is not difficult, a learning curve was observed. The probe needed to be very lightly applied with generous amounts of contact jelly otherwise artefactual areas of stiffness radiating from the skin surface were produced. The probe needs to be kept still for 10 to 20 seconds during acquisition of the elastography images (due to a slow frame rate), and this was often best done during a breath hold. The maximum areas of stiffness in malignant cases was almost always found in the peri-tumoural stroma rather than in the cancer itself, so it was important to make sure these peri-tumoural areas were adequately imaged. The elastography views selected were those most clearly displaying abnormal stiffness within the plane but with the absence of movement or pressure artefact. In a subset of 15 patients, an additional observer produced pairs of elastography images (giving four images in total). Twenty women with normal clinical and ultrasound examinations also underwent elastography.

Two independent observers recorded the maximum stiffness, mean stiffness and SD within a ROI placed in the stiffest areas on the colour maps on both of the two elastography images obtained. As the ROI is moved around the image with a cursor, the elastography values are displayed instantaneously in a data box to the side of the image, allowing the ROI to be placed in the area of greatest stiffness on the image. The average values from the two images were used for analysis. The mean elasticity values from each pair of images were then compared between observers. In the 15 cases where a second pair of images was acquired by a different observer, values from the first pair were used for the main analyses - but the mean elasticity value produced by the first observer was additionally compared with that produced by the second observer, and the correlation coefficient was calculated.

After preliminary experience with 10 patients, cut-off values for mean elasticity (50 kPa), for maximum elasticity (55 kPa) and for the SD (10 kPa) were selected for benign/malignant differentiation on shear wave elastography. The preliminary experience of 10 patients included the following lesions: three fibroadenomas, one benign papilloma, one mass forming DCIS and five invasive ductal carcinomas. The 50 kPa cut-off value for mean elasticity was chosen because the highest value obtained from a benign lesion (the papilloma) was 49 kPa and the lowest value from a cancer was 56 kPa. The mean lesion size of these patients was 19 mm.

Benign/malignant differentiation on greyscale ultrasound with BI-RADS classification and shear wave elastography using the defined cut-off values were compared with histology to provide figures for sensitivity, specificity, positive predictive value (PPV), negative predictive value (NPV) and accuracy. In addition, the area under the ROC curve was calculated for both modalities. These performance criteria were then compared between greyscale BI-RADS and shear wave elastography. Histological findings from surgery if performed, and otherwise from core biopsy, were used as the gold standard. Invasive cancer, DCIS and lobular carcinoma *in situ *were counted as malignant.

### Statistical analyses

Intraclass correlation coefficients using web-based software [18], Fisher's exact tests and ROC analyses (with associated measures, including sensitivity, specificity and accuracy) were used to assess the equivalence of greyscale BI-RADS with shear wave elastography. The null hypothesis was rejected at an α level of 5% (*P *≤ 0.05).

## Results

Fifty-two women with 53 solid breast masses comprised the study group. The age range was 18 to 84 years, and the mean age was 53 years. Forty-seven lesions were symptomatic while six lesions were detected at mammographic screening. The mean ultrasound size of the symptomatic lesions was 21 mm and the screening detected lesions of 12 mm. The BI-RADS scores were: BI-RADS 2, two lesions (4%); BI-RADS 3, 20 lesions (38%); BI-RADS 4, 15 lesions (28%); and BI-RADS 5, 16 lesions (30%). Histology revealed 23 benign lesions and 30 cancers (28 invasive cancers, one DCIS and one lobular carcinoma *in situ*) in the study group.

### Reproducibility

**Figure 1:**
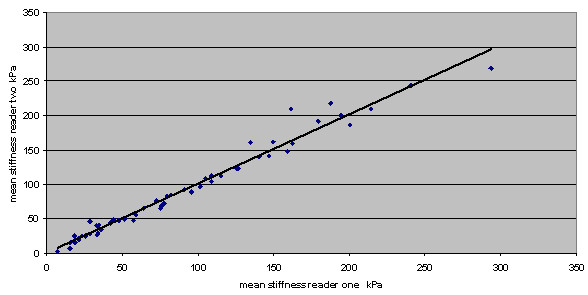
**Correlation of mean stiffness measurements by two independent observers on each pair of elastography images**. *r *= 0.99.

The correlation between measurements by two independent observers of the mean stiffness on each pair of elastography images acquired by a single operator is shown in Figure 1. The intraclass correlation coefficient is 0.99, indicating that deciding which area on an image has the highest mean stiffness and measuring this area was highly reproducible. Three of 53 patients (6%) fell into different benign/malignant classifications when comparing the two observers. These three cases had mean stiffness values close to the 50 kPa cut-off value. The average difference in mean stiffness between the observers in these three discordant cases was 6 kPa. Intraclass correlation coefficients between two observers for maximum stiffness and SD, averaged from two images, were 0.98 and 0.93, respectively.

**Figure 2:**
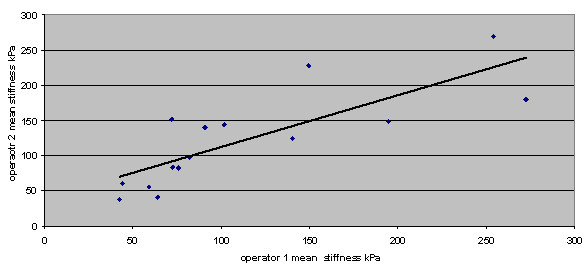
**Correlation of mean stiffness measurements on pairs of elastography images taken by two independent operators**. *r *= 0.80.

The correlation between measurements of the mean stiffness on pairs of elastography images acquired and read by two independent operators is shown in Figure 2. The intraclass correlation coefficient was 0.80, indicating moderate agreement. In two (13%) of the 15 patients the stiffness measurements taken from the images taken by two operators fell into nonconcordant benign/malignant classifications.

### Normal tissue

The elastography parameters of the 20 women with normal clinical and ultrasound findings are presented in Table 1. This indicates that normal tissue has low mean and maximum stiffness. The low SD values indicate low levels of tissue heterogeneity. Stiffness measurements of fatty and parenchymal tissue were not obviously different.

**Table 1:** Age and elastography parameters of 20 women with normal physical and greyscale ultrasound examinations

	Lowest value	Highest value	Mean
Age	13	66	44
Mean stiffness	7	35	22
Maximum stiffness	9	45	28
Standard deviation	1	33	5

### Benign lesions

**Figure 3:**
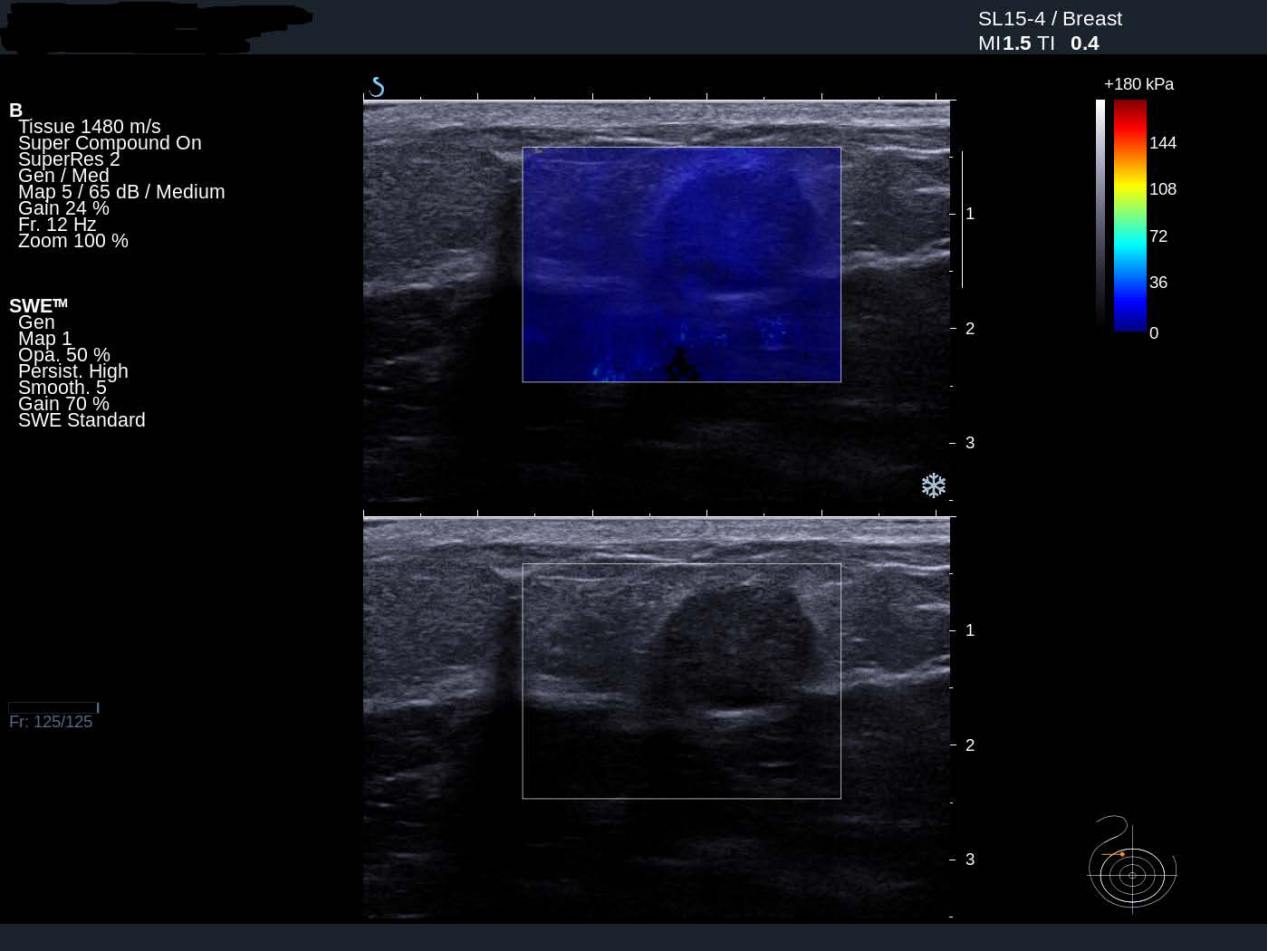
**Ultrasound and elastography images of a fibroadenoma showing low stiffness**. Mean 25 kPa.

**Figure 4:**
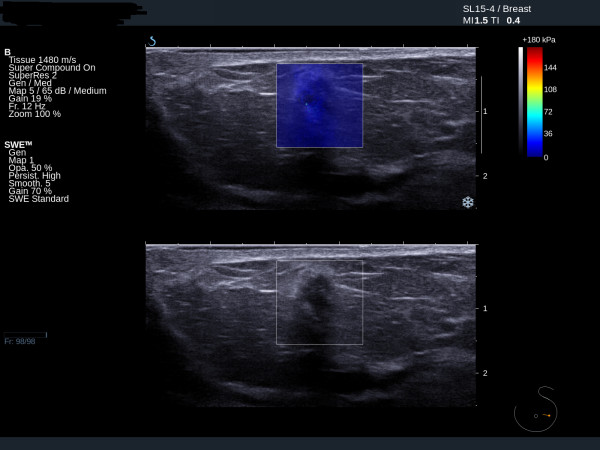
**Ultrasound and elastography images of a benign fibroadenoma**. Ultrasound and elastography images showing a benign fibroadenoma with suspicious greyscale ultrasound features but benign elastography features. Mean 32 kPa.

Fibroadenomas show low mean stiffness (average 28 kPa, range 18 to 44 kPa), maximum stiffness and SD values (Figures 3 and 4). The four benign lesions with mean stiffness above the 50 kPa threshold were two radials scars (both BI-RADS 4), one fat necrosis (BI-RADS 4) and one cellular fibroepithelial lesion (BI-RADS 3) removed to exclude a phyllodes tumour. Three of the four benign lesions with high elasticity values had core biopsy results showing lesions of uncertain malignant potential. The two cases of radial scar and the fat necrosis case were also classified as either BI-RADS 4 or BI-RADS 5 on greyscale imaging.

### Ductal carcinoma *in situ*

The one DCIS lesion had a mean stiffness in the lower part of the malignant range (76 kPa). This lesion was a mass classified as BI-RADS 3.

### Lobular carcinoma *in situ*

The single lobular carcinoma *in situ *case was in a mammographic distortion with malignant BI-RADS and elastographic features (mean stiffness, 82 kPa).

### Invasive cancer

**Figure 5:**
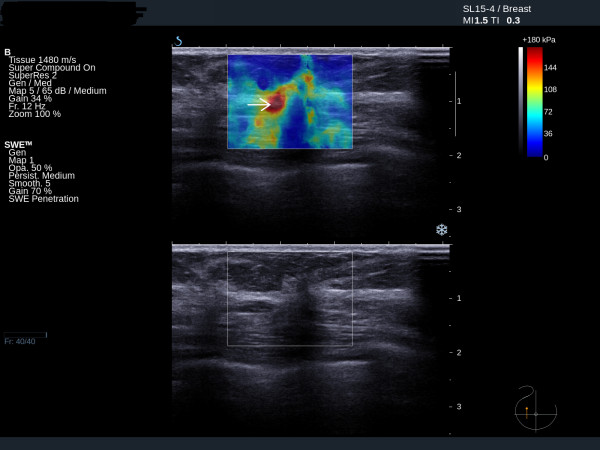
**Ultrasound and elastography images of an invasive ductal cancer**. Ultrasound and elastography images of an invasive ductal cancer showing typical peri-tumoural stiffness (arrow).

**Figure 6:**
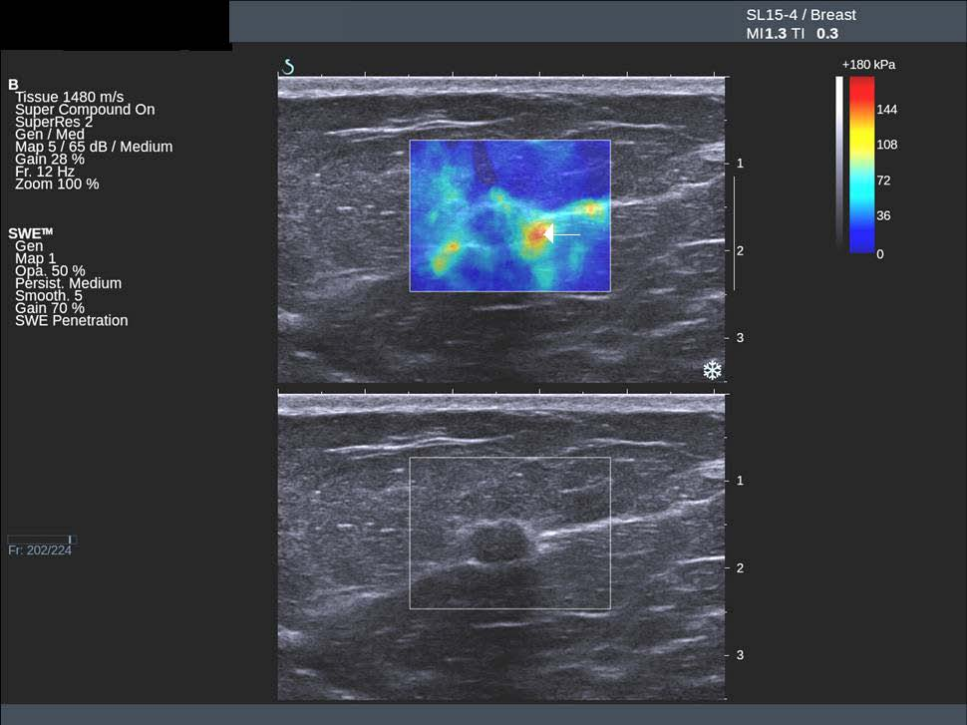
**Ultrasound and elastography images of an invasive cancer**. Ultrasound and elastography images showing an invasive cancer with benign greyscale features but suspicious peri-tumoural stiffness on the elastography image (arrow).

Twenty-seven (96%) of the 28 invasive cancers had mean elasticity values above the 50 kPa threshold (Figures 5 and 6). The average mean value was 140 kPa (range 29 to 293 kPa). Invasive cancers with an ultrasound size <15 mm had an average mean elasticity of 109 kPa, compared with an average value for lesions ≥ 15 mm of 167 kPa. The lesion with a mean stiffness below the 50 kPa threshold was a 12 mm grade 2 invasive ductal carcinoma of no special type, which was classified as BI-RADS 3. The lesion was sited very high in the breast and abutted both the skin and the pectoral muscle with very little surrounding breast tissue. The malignant lesions misclassified by greyscale ultrasound were a single DCIS case and three cases of invasive ductal carcinoma.

### Benign/malignant differentiation

Shear wave elastography performance for maximum elasticity was sensitivity 97%, specificity 78%, PPV 85%, NPV 95% and accuracy 89%. For the SD, the performance was sensitivity 83%, specificity 96%, PPV 96%, NPV 95% and accuracy 89%. The number of true positive results, false positive results, true negative results and false negative results for each parameter are shown in Table 2.

**Table 2:** Numbers of lesions in each group according to greyscale BI-RADS and shear wave elastography parameters

Results	BI-RADS	Mean stiffness	Maximum stiffness	Standard deviation
True positive	26	29	29	25
False positive	5	4	5	1
True negative	18	19	18	22
False negative	4	1	1	5

BI-RADS, Radiology Breast Imaging and Reporting Data System.

**Figure 7:**
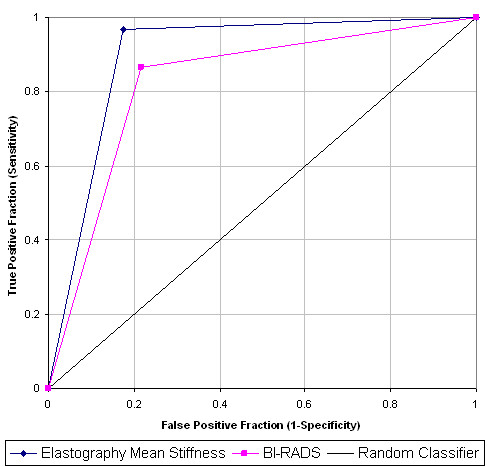
**Receiver-operating characteristic curve comparing performance**. Receiver-operating characteristic curve to compare the performance of greyscale Breast Imaging and Reporting Data System (BI-RADS) with shear-wave elastography mean stiffness.

Strong associations were demonstrated between greyscale BI-RADS findings, shear wave elastography findings (mean elasticity, maximum elasticity and SD) and histology (*P *< 0.0001 for all: Fisher's exact test). For mean elasticity versus greyscale BI-RADS, the performance results were sensitivity 97% versus 87%, specificity 83% versus 78%, PPV 88% versus 84%, NPV 95% versus 82% and accuracy 91% versus 83% (Table 3). The performance of shear wave elastography outperformed that of greyscale BI-RADS in all standard aspects of performance, including accuracy, PPV, NPV, area under the curve on a ROC analysis (Figure 7), and Youden's Index; however, this difference did not reach statistical significance (*P *= 0.129, Mann-Whitney U test).

**Table 3:** Performance results of mean tissue elasticity versus greyscale BI-RADS

	Elastography	BI-RADS
Positive predictive value (%)	88 (77 to 99)	84 (71 to 97)
Negative predictive value (%)	95 (85 to 100)	82 (66 to 98)
Sensitivity (true positive fraction) (%)	97 (90 to 100)	87 (75 to 99)
Specificity (true negative fraction) (%)	83 (67 to 98)	78 (61 to 95)
Accuracy (%)	91	83
Misclassification rate (%)	9	17
Youden's index	0.79	0.65
Area under curve	0.90 (0.81 to 0.98)	0.83 (0.71 to 0.94)
Area under curve difference	0.07 (-0.02 to 0.16)	
*P *value (Mann-Whitney U test)	0.129	

Data presented as mean (95% confidence interval). BI-RADS, American College of Radiology Breast Imaging and Reporting Data System.

## Discussion

We have shown that several shear wave elastography parameters are useful in aiding benign/malignant differentiation of solid breast masses. Shear wave elastography differs from conventional elastography in that it provides quantitative data and appears to be highly reproducible. The most useful shear wave measure appears to be the mean stiffness within a ROI placed on the stiffest part of a saved image. A mean stiffness of over 50 kPa is highly suggestive of malignancy. Maximum and stiffness SD values show diagnostic accuracy and reproducibility almost as good as mean stiffness. As the electronic cursor delineating the ROI was moved to the area giving the highest mean elasticity value and the maximum and SD readings were taken from this position, it is possible that positioning the cursor according to highest maximum and SD values would further improve their diagnostic value. SD is likely to be of value in benign/malignant differentiation because it is a measure of lesion heterogeneity, which is more common and more marked in malignant lesions compared with benign lesions. Heterogeneity has been used to aid benign/malignant differentiation using greyscale ultrasound for many years [3]. In view of the whole dataset, the cut-off value of 50 kPa gives a good balance of high sensitivity and specificity. The choice of cut-off value is also dependent on what use will be made of the data. To increase the number of benign breast lesions that do not need to undergo biopsy, one might sacrifice specificity to some degree to increase sensitivity; that is, adopt a lower threshold value. On average, small cancers were not as stiff as larger cancers but not to the extent of bringing the stiffness values down to the benign range.

The findings of our study regarding the ability of mean elasticity values to differentiate between benign and malignant lesions are similar to those of Athanasiou and colleagues [15]. There are, however, a number of differences between that study and our study. The previous study included only lesions that were mammographically occult, whereas the current study involved a broad range of lesions including some lesions detected on mammographic screening. In the previous study, the greyscale imaging was done with a different machine to that producing the elastography. In the current study, greyscale and elastography imaging were performed on the same machine. The previous study was performed on a prototype that required offline computer post processing, which produced a single lesional elasticity value. The current study was performed on a production model with immediate production of colour maps and quantitative values. Three different values were evaluated in the present study (maximum and mean elasticity and SD). Two sets of values taken from different imaging planes were obtained and the values averaged. The previous study made no attempt to address interobserver variability where we have addressed two possible sources of such variability.

Careful evaluation of what the peri-tumoural stiffness seen at elastography represents histologically has not been performed. Some authors have suggested this might represent surrounding DCIS, while others suggest the desmoplastic reaction associated with many breast cancers may be responsible. Further authors have suggested it might be invasive tumour infiltration too small to be seen by conventional ultrasound imaging [7,9]. This last suggestion is unlikely to be true since ultrasound has been shown to predict whole invasive tumour size accurately in most cases. This would not be so if the stiff areas surrounding cancers that appear normal on greyscale ultrasound represent small foci of invasive cancer.

In the present study, normal tissue and fibroadenomas had uniformly low elasticity values. The benign lesions that were stiff tended to be lesions of uncertain malignant potential such as radial scars. The other nonmalignant lesions that we have found anecdotally to be stiff but were not included in this study, as they were not focal solid lesions, include peri-abscess inflammation, surgical scars and thickened skin following radiotherapy.

The average values obtained by manipulation of the electronic cursor within pairs of elastography images to find the stiffest region were highly reproducible (intraclass correlation coefficient 0.99). Even the stiffness measurements obtained from pairs of images taken by different operators (intraclass correlation coefficient 0.80) show good reproducibility. We think it highly likely that improved correlation between different operators could be achieved if more elastography images were obtained from each lesion; for example, four images rather than the two obtained in the present study.

Use of BI-RADS [16] categorisation of ultrasound images is widespread in the USA but utilised less in Europe. The BI-RADS 4 category includes all lesions with a 3 to 95% chance of being malignant. Lesions with typical benign appearances (BI-RADS 3) are routinely placed on short-term follow-up in the USA, but most such lesions in women over 25 years old would undergo core biopsy in Europe. The BI-RADS categorisation was used in the present study as a comparator for shear wave elastography because it is the most widely used and recognised classification of breast masses on ultrasound internationally.

The present study suggests that elastography classification is at least as accurate as BI-RADS in separating benign and malignant lesions, but this requires confirmation. This study has a number of limitations. This was a small, single-centre study, and the numbers of observers and cancers were small - in particular, only one DCIS case was included. It is important that similar studies are performed in multiple centres on a large number of patients with symptomatic and screen-detected masses. If our findings are replicated, the next step would be to determine how best to combine shear wave elastography and greyscale ultrasound findings to enhance benign/malignant differentiation.

## Conclusions

Shear wave elastography gives quantitative and reproducible information on solid breast lesions with diagnostic accuracy at least as good as greyscale ultrasound with BI-RADS classification. After further work, it might be possible to increase the proportion of women with benign masses who can be reassured and discharged based on the ultrasound findings without recourse to ultrasound-guided core biopsy.

## Abbreviations

BI-RADS: Breast Imaging and Reporting Data System; DCIS: ductal carcinoma *in situ*; NPV: negative predictive value; PPV: positive predictive value; ROC: receiver-operating characteristic; ROI: region of interest; SD: standard deviation.

## Competing interests

The authors declare that they have no competing interests.

## Authors' contributions

AE was involved in the study design, data acquisition and data analysis, and drafted the manuscript. PW was involved in the study design and edited the manuscript. KT was involved in data acquisition and edited the manuscript. DM was involved with data acquisition and edited the manuscript. KB was involved with BI-RADS classification and edited the manuscript. CP was involved in the study design and edited the manuscript. LJ was involved in the study design and edited the manuscript. LB was involved in the statistical analysis. AT was involved in the study design and edited the manuscript. All authors read and approved the final manuscript.
